# A regulatory BMI1/let‐7i/ERK3 pathway controls the motility of head and neck cancer cells

**DOI:** 10.1002/1878-0261.12021

**Published:** 2017-01-12

**Authors:** Lobna Elkhadragy, Minyi Chen, Kennon Miller, Muh‐Hwa Yang, Weiwen Long

**Affiliations:** ^1^ Department of Biochemistry and Molecular Biology Boonshoft School of Medicine Wright State University Dayton OH USA; ^2^ Department of Pathology Boonshoft School of Medicine Wright State University Dayton OH USA; ^3^ Institute of Clinic Medicine National Yang‐Ming University Taipei Taiwan

**Keywords:** BMI1, cell migration, ERK3, head and neck cancer, let‐7i

## Abstract

Extracellular signal‐regulated kinase 3 (ERK3) is an atypical mitogen‐activated protein kinase (MAPK), whose biological activity is tightly regulated by its cellular abundance. Recent studies have revealed that ERK3 is upregulated in multiple cancers and promotes cancer cell migration/invasion and drug resistance. Little is known, however, about how ERK3 expression level is upregulated in cancers. Here, we have identified the oncogenic polycomb group protein BMI1 as a positive regulator of ERK3 level in head and neck cancer cells. Mechanistically, BMI1 upregulates ERK3 expression by suppressing the tumor suppressive microRNA (miRNA) let‐7i, which directly targets ERK3 mRNA. ERK3 then acts as an important downstream mediator of BMI1 in promoting cancer cell migration. Importantly, ERK3 protein level is positively correlated with BMI1 level in head and neck tumor specimens of human patients. Taken together, our study revealed a molecular pathway consisting of BMI1, miRNA let‐7i, and ERK3, which controls the migration of head and neck cancer cells, and suggests that ERK3 kinase is a potential new therapeutic target in head and neck cancers, particularly those with BMI1 overexpression.

**Abbreviations**
ERK3: extracellular signal‐regulated kinase 3
HNSCC: head and neck squamous cell carcinoma
MAPK: mitogen‐activated protein kinase
miRNA: microRNA
MMP: matrix metalloproteinase
PRC1: polycomb repressive complex‐1
SRC‐3: steroid receptor coactivator 3

## 1 Introduction

Dysregulation of signal transduction pathways is a hallmark of many cancers (Cargnello and Roux, 2012; Lei *et al*., 2014). While the implication of several conventional mitogen‐activated protein kinase (MAPK) pathways in cancers is well studied, the involvement of the atypical MAPKs in tumorigenesis is poorly understood (Kostenko *et al*., 2012). Extracellular signal‐regulated kinase 3 (ERK3), also known as MAPK6, is an atypical member of the MAPK family (Coulombe and Meloche, 2007; Kostenko *et al*., 2012). The importance of ERK3 signaling in cancers has been recently recognized following our previous finding that ERK3 promotes cancer cell invasiveness by phosphorylating steroid receptor coactivator 3 (SRC‐3) oncoprotein and upregulating SRC‐3‐mediated transcription of matrix metalloproteinase (MMP) genes (Long *et al*., 2012). In addition, ERK3 was shown to promote breast cancer cell migration by regulating cell morphology and spreading (Al‐Mahdi *et al*., 2015). Furthermore, ERK3 enhances the activity of tyrosyl DNA phosphodiesterase 2 (TDP2) in DNA damage response and increases the chemoresistance of lung cancer cells to topoisomerase‐2 inhibitors (Bian *et al*., 2016). In line with its important roles in cancer cell migration, invasion, and DNA damage response, ERK3 is upregulated in multiple cancers, including non‐small‐cell lung cancer (Long *et al*., 2012), gastric cancer (Liang *et al*., 2005), and oral squamous cell carcinoma (Rai *et al*., 2004). Little is known, however, about the molecular mechanisms of ERK3 upregulation in cancers. The level of ERK3 protein in cells is thought to be a critical regulator for ERK3 activity, as unlike other MAPK family members, ERK3 is a highly unstable protein with a half‐life of 30‐45 minutes in exponentially proliferating cells (Coulombe *et al*., 2003, 2004).

BMI1 is a key regulatory component of the transcription suppressor complex, the polycomb repressive complex‐1 (PRC1) (Cao *et al*., 2011; Siddique and Saleem, 2012). It plays important roles in the maintenance and self‐renewal of normal and cancer stem cells (Lessard and Sauvageau, 2003; Park *et al*., 2003; Rizo *et al*., 2009; Schuringa and Vellenga, 2010) and promotes tumor cell growth, migration, and invasion, thereby promoting tumor growth and progression (Cao *et al*., 2011; Jiang *et al*., 2009; Siddique and Saleem, 2012; Wu *et al*., 2011). BMI1 functions as an oncoprotein by silencing various tumor suppressor genes, such as p16Ink4a, p14Arf, PTEN (Cao *et al*., 2011; Jacobs *et al*., 1999; Song *et al*., 2009), and microRNAs (miRNAs) including let‐7i (Chou *et al*., 2013; Yang *et al*., 2012). miRNAs act as post‐transcriptional regulators of gene expression by repressing mRNA translation and/or facilitating mRNA degradation (Lee, 2014; Ranganathan and Sivasankar, 2014). Recent studies have shown that let‐7i plays tumor suppressive roles by inhibiting tumor cells’ growth and migration (Fawzy *et al*., 2016; Subramanian *et al*., 2015; Tian *et al*., 2015; Wu *et al*., 2015, 2016; Yang *et al*., 2012; Zhang *et al*., 2015). let‐7i is shown to be downregulated in several cancers including head and neck squamous cell carcinomas (HNSCCs; Liu *et al*., 2012; Roush and Slack, 2008; Subramanian *et al*., 2015; Yang *et al*., 2012). HNSCC patients with lower levels of let‐7i had increased local invasion of tumor cells to adjacent tissues (Yang *et al*., 2012).

In this study, we revealed a molecular mechanism for the regulation of ERK3 expression in head and neck cancer cells: BMI1 upregulates ERK3 by suppressing let‐7i miRNA that directly targets ERK3 mRNA. Importantly, our study reveals a regulatory pathway consisting of BMI1, let‐7i, and ERK3 that is important for controlling cancer cell migration.

## 2 Material and methods

### 2.1 Cell culture

The human oral cancer cell line OECM‐1 was maintained in RPMI 1640 medium supplemented with 10% fetal bovine serum (FBS). The following human cell lines were cultured in Dulbecco's modified Eagle medium supplemented with 10% FBS: Fadu (hypopharyngeal cancer), UMSCC1 (oral cavity cancer), Detroit‐562 (metastatic pharyngeal), 293T (embryonic kidney), and HeLa (cervical cancer). All the culture media and supplements were purchased from Gibco/ThermoFisher Scientific (Waltham, MA, USA).

### 2.2 Expression plasmids

The lentiviral expression construct of BMI1 with a HA tag at the N terminus (pCDH‐BMI1) was generated by inserting the HA‐BMI1 fragment released from pT3‐EF1a‐Bmi1 by *Asc*I/*Sac*II digestion into pCDH‐CMV‐MCS‐EF1‐Puro (System Biosciences, Palo Alto, CA, USA) digested with SwaI. The lentiviral expression construct of ERK3 with 6 Myc tags at the N terminus (pCDH‐Myc6‐ERK3) was generated as described previously (Long *et al*., 2012).

Luciferase reporter constructs containing a luciferase gene upstream of either a random control 3′ untranslated region (3′UTR) or the entire 3′UTR of ERK3 were obtained from SwitchGear Genomics (Carlsbad, CA, USA) and designated as pLightSwitch‐ctrl‐3′UTR and pLightSwitch‐ERK3‐3′UTR, respectively. A luciferase reporter with mutated ERK3 3′UTR (pLightSwitch‐ERK3‐mt3′UTR) in which seven nucleotides in the let‐7i binding site of ERK3 3′UTR (487: 5′‐CUACCUC) were replaced by AGCAAGA nucleotides was generated by site‐directed mutagenesis using QuikChange II XL Site‐Directed Mutagenesis Kit (Agilent Technologies, Santa Clara, CA, USA) and the following primers: 5′‐gttttgccacatactcttgttacctttcttgctataacacatgtgtaccaaattcggcattcattttcagttgctgctg‐3′ and 5′‐cagcagcaactgaaaatgaatgccgaatttggtacacatgtgttatagcaagaaaggtaacaagagtatgtggcaaaac‐3′. The sequence of the resulting mutant plasmid was verified by sequencing.

### 2.3 Generation of stable cell pools expressing shRNA or cDNA

OECM1 cell line with stable knockdown of BMI1 (OECM1‐shBMI1) was generated by lentiviral expression of a short hairpin RNA (shRNA) specifically targeting BMI1 (shBMI1) as described previously (Yang *et al*., 2012). OECM1 cells with stable expression of a scrambled nontargeting shRNA (OECM1‐shCtrl) served as a control. Fadu cells with stable overexpression of BMI1 (Fadu‐BMI1) or the control empty vector pCDH‐CMV‐MCS‐EF1‐Puro (Fadu‐CDH) were generated as follows. First, pseudotyped lentiviral particles were produced in 293T cells by cotransfecting pCDH‐BMI1 or pCDH‐CMV‐MCS‐EF1‐Puro with Trans‐Lentiviral Packaging Plasmid Mix (Open Biosystems, Lafayette, CO, USA). Pseudoviral particles were harvested 48 h after transfection and concentrated using PEG‐it Virus Precipitation Solution (System Biosciences), following the manufacturer's instructions. Next, Fadu cells were transduced with the prepared virus in the presence of polybrene (4 μg·mL^−1^). Two days post‐transduction, cells were split and selected by puromycin (1 μg·mL^−1^) for 10 days. The stable overexpression of BMI1 was verified by western blotting analysis.

### 2.4 Transient lentiviral transduction

OECM1 stable cell pools were transiently transduced with lentiviruses expressing either an empty vector pCDH or pCDH‐Myc6‐ERK3 in the presence of 4 μg·ml^−1^ polybrene for 2 days.

### 2.5 Transient transfection

Transient transfections with plasmids were performed using Lipofectamine 3000 Reagent (Invitrogen/ThermoFisher Scientific, Carlsbad, CA, USA), whereas transient transfections with siRNAs, miRNA mimics, or miRNA inhibitors were performed using DharmaFECT Transfection Reagent (Dharmacon, Lafayette, CO, USA), following the manufacturer's instructions. BMI1 siRNA ON‐TARGET Plus SMART pool and nontargeting control siRNA pool were purchased from Dharmacon. The silencer select siRNA targeting human ERK3 and the Silencer Negative Control #1 were purchased from Ambion/ThermoFisher Scientific (Waltham, MA, USA). The following miRNA mimics and inhibitors were purchased from Dharmacon: miRIDIAN microRNA mimic negative control #1, miRIDIAN hsa‐let‐7i‐5p mimic, miRIDIAN microRNA hairpin inhibitor negative control #1, and miRIDIAN hsa‐let‐7i‐5p hairpin inhibitor.

### 2.6 Western blotting

Cells were lysed with EBC lysis buffer [50 mm Tris (pH 7.5), 150 mm NaCl, 0.5% NP‐40, 1 mm Complete protease inhibitors (Roche Diagnostics, Basel, Switzerland), and 1 mm phosphatase inhibitor cocktail III (Sigma‐Aldrich, St. Louis, MO, USA)]. Western blotting was performed by following the procedures as described previously (Long *et al*., 2012) with the use of the following primary antibodies: anti‐ERK3 (Abcam, Cambridge, UK), anti‐BMI1 (Cell Signaling, Danvers, MA, USA), and anti‐β‐actin (Sigma‐Aldrich). β‐Actin was used as a loading control in the western blotting.

### 2.7 RNA extraction and RT‐qPCR

For gene expression analysis, the total RNA was extracted from cells using Trizol reagent (Ambion, Waltham, MA, USA) and reverse transcription (RT) was carried out using SuperScript VILO Master Mix (Invitrogen, Carlsbad, CA, USA) according to the manufacturer's protocol. Quantitative polymerase chain reaction (qPCR) was performed using TaqMan Probe system (Roche Diagnostics) on the Applied Biosystems 7500 (Applied Biosystems/ThermoFisher Scientific, Foster City, CA, USA) with GAPDH as the internal control. Relative expression to normalizer sample was calculated using the ΔΔCT method.

To measure miRNA expression, total RNA was extracted using mirVana miRNA Isolation Kit (Ambion) and reverse‐transcribed to cDNA using TaqMan Advanced MicroRNA cDNA Synthesis Kit (Applied Biosystems/ThermoFisher Scientific, Foster City, CA, USA). qPCR was performed using TaqMan Advanced MicroRNA Assay kit (ThermoFisher Scientific, Waltham, MA, USA) following the manufacturer's instructions. The relative expression of hsa‐let‐7i‐5p was normalized to that of hsa‐miR‐191‐5p.

### 2.8 Luciferase reporter assay

HeLa cells were cotransfected with a luciferase reporter plasmid and a miRNA mimic or inhibitor. The luciferase activity was measured 30 h or 44 h, respectively, post‐transfection using LightSwitch Luciferase Assay Kit (SwitchGear Genomics).

### 2.9 Two‐chamber transwell cell migration assay

Cell migration was analyzed using a modified two‐chamber transwell system (BD Biosciences, San Jose, CA, USA), following the manufacturer's instructions. Cells were detached by trypsin/EDTA, washed once with serum‐free medium, and then resuspended in medium with 2% FBS (for OECM1 cells) or serum‐free medium (for Fadu cells). Complete culture medium with 10% FBS was added to each bottom well. Cells were added in transwell inserts and allowed to migrate for 18 h (for OECM1 cells) or 30 h (for Fadu cells) in a 37 °C cell incubator. The cells in the upper surface of the transwell were removed using cotton swabs and migrated cells attached on the undersurface were fixed with 4% paraformaldehyde for 10 min and stained with crystal violet solution (0.5% in water) for 10 min. Migrated cells were then photographed and counted under a microscope at 50 × magnification.

### 2.10 Wound healing assay

Wound healing was assayed in confluent OECM1 cell monolayers. Cells were scraped using a standard 200‐μL pipette tip and the wounded monolayers were washed twice to remove nonadherent cells. Cells were photographed every 5 h, and the wound width was measured. Wound closure was calculated by subtracting the wound width at 20‐h time point from the initial wound width (mm).

### 2.11 Immunohistochemical analysis of ERK3 and BMI1 in tumor tissue microarrays

To determine the expression of ERK3 and BMI1 proteins in head and neck cancer tissues, we purchased two identical head and neck cancer tissue microarrays (HN803c; US Biomax, Derwood, MD, USA). Each microarray was comprised of the following formalin‐fixed, paraffin‐embedded (FFPE) tissues: 60 cases of squamous cell carcinoma of larynx, palate, lower lip, upper jaw, tongue, gingiva, larynx, throat, mandible, or cheek and eight cases of metastatic carcinoma. ERK3 and BMI1 proteins were immunostained following the procedures described previously (Cai *et al*., 2010) with the following modifications. Antigen retrieval was performed by treating the slides in citrate‐based antigen unmasking solution (pH 6.0; Vector Laboratories, Burlingame, CA, USA) at 90 °C for 12 min using a pressure cooker. One slide was incubated with an antibody against ERK3 (1:50 dilution; Abcam) and the other slide was incubated with an antibody against BMI1 (1 : 400; Cell Signaling) overnight at 4 °C, followed by the fluorescent labeling with Alexa Fluor 488 anti‐rabbit secondary antibody (Invitrogen) and DAPI staining. The tissues were visualized and imaged using a Leica CTR 6000 Microscope (Leica Microsystems, Wetzlar, Germany) and imagepro 6.2 software (Media Cybernetics, Rockville, MD, USA). Tumor lesions in each sample were identified by examining an H&E‐stained slide with the same tumor tissue samples. Multiple measurements of the fluorescent staining intensity of ERK3 and BMI1 in tumor regions (at least three fields) for each sample were made using imagepro 6.2 software and were normalized by subtracting background fluorescence intensity.

### 2.12 Statistics

Data are expressed as mean ± standard deviation (SD) or standard error (SE) as specified in the figure legends. Statistical significance was determined by a two‐sided Student's *t*‐test, where a *P*‐value of less than 0.05 was considered statistically significant. The correlation between ERK3 and BMI1 mean fluorescence intensities was determined using the Pearson's correlation analysis and *P* = 0.001.

## 3 Results

### 3.1 BMI1 upregulates the expression of ERK3 in head and neck cancer cells

**Figure 1:**
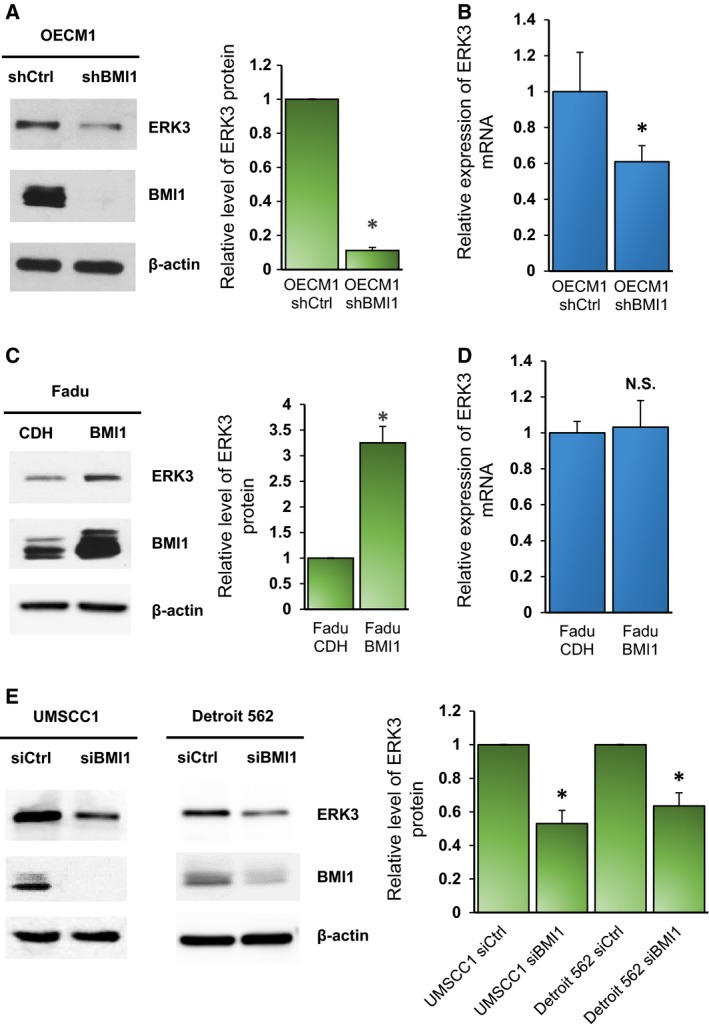
BMI1 upregulates ERK3 expression in head and neck cancer cells. (A) Left panel: western blot analysis of ERK3 and BMI1 in OECM1 cells with stable expression of a nontargeting scrambled shRNA (OECM1‐shCtrl) or a shRNA specifically targeting BMI1 (OECM1‐shBMI1). Right panel: quantification of ERK3 protein level in stable OECM1 cells by densitometry analysis of the immunoblots using imagej software (National Institutes of Health, Bethesda, Maryland, USA). The band density in ‘shCtrl’ was set as 1. The bar graph represents the mean ± SE of three independent experiments, *P < 0.001 (Student's *t*‐test). (B) ERK3 gene expression analysis by RT‐qPCR in OECM1‐shCtrl and OECM1‐shBMI1 cells. Values in bar graphs represent mean ± SD of three experiments. *P < 0.05 (Student's *t*‐test). (C) Left panel: western blot analysis of ERK3 and BMI1 in Fadu cells with stable expression of an empty vector (Fadu‐CDH) or BMI1 (Fadu‐BMI1). Right panel: quantification of ERK3 protein level in stable Fadu cells by densitometry analysis of the immunoblots. The band density in ‘CDH’ was set as 1. The bar graph represents the mean ± SE of three experiments. *P < 0.01 (Student's *t*‐test). (D) RT‐qPCR analysis of ERK3 gene expression in stable Fadu cells. Values in bar graphs represent mean ± SD of three experiments. NS, not significant (Student's *t*‐test). (E) Left panel: western blot analysis of ERK3 and BMI1 protein levels in UMSCC1 and Detroit 562 head and neck cancer cells with transient transfection of a nontargeting control siRNA (siCtrl) or a siRNA against BMI1 (siBMI1). Right panel: quantification of ERK3 protein level in the immunoblots. The band density in ‘siCtrl’ was set as 1. Data represent mean ± SE of three independent experiments, *P < 0.05 (Student's *t*‐test).

To elucidate the regulation of ERK3 expression in cancers, we attempted to search databases and publications for potential regulators of ERK3. Interestingly, a recent study of transcriptional profiling in a multiple myeloma cell line with stable knockdown of BMI1 showed that ERK3 is among the downstream target genes of BMI1 (Jagani *et al*., 2010). As BMI1 is an oncogenic transcriptional factor for cancer cell growth and migration (Cao *et al*., 2011) and both BMI1 and ERK3 are overexpressed in head and neck cancers (Rai *et al*., 2004; Song *et al*., 2006), we were interested in investigating the molecular regulation of ERK3 by BMI1 and the functional significance of this axis in head and neck cancer cells. A previous study showed that as compared to Fadu cells, OECM1 cells have a higher endogenous level of BMI1 and are more migratory (Chou *et al*., 2013). Hence, we examined ERK3 levels in OECM1 cells after stable knockdown of BMI1 and in Fadu cells with stable BMI1 overexpression. Stable knockdown of BMI1 in OECM1 cells (OECM1‐shBMI1) greatly reduced ERK3 protein level (Fig. 1A), whereas BMI1 overexpression in Fadu cells increased ERK3 protein level (Fig. 1C). Interestingly, while ERK3 mRNA level was significantly decreased upon BMI1 knockdown in OECM1 cells (Fig. 1B), BMI1 overexpression in Fadu cells did not alter ERK3 mRNA expression (Fig. 1D). The positive regulation of ERK3 expression by BMI1 was confirmed in two other head and neck cancer cell lines, UMSCC1 and Detroit 562, in which transient knockdown of BMI1 by siRNA (siBMI1) led to a decrease in ERK3 protein level (Fig. 1E). Taken together, these results demonstrate that BMI1 positively regulates ERK3 expression in head and neck cancer cells.

### 3.2 BMI1 regulates ERK3 by an indirect mechanism involving let‐7i

**Figure 2:**
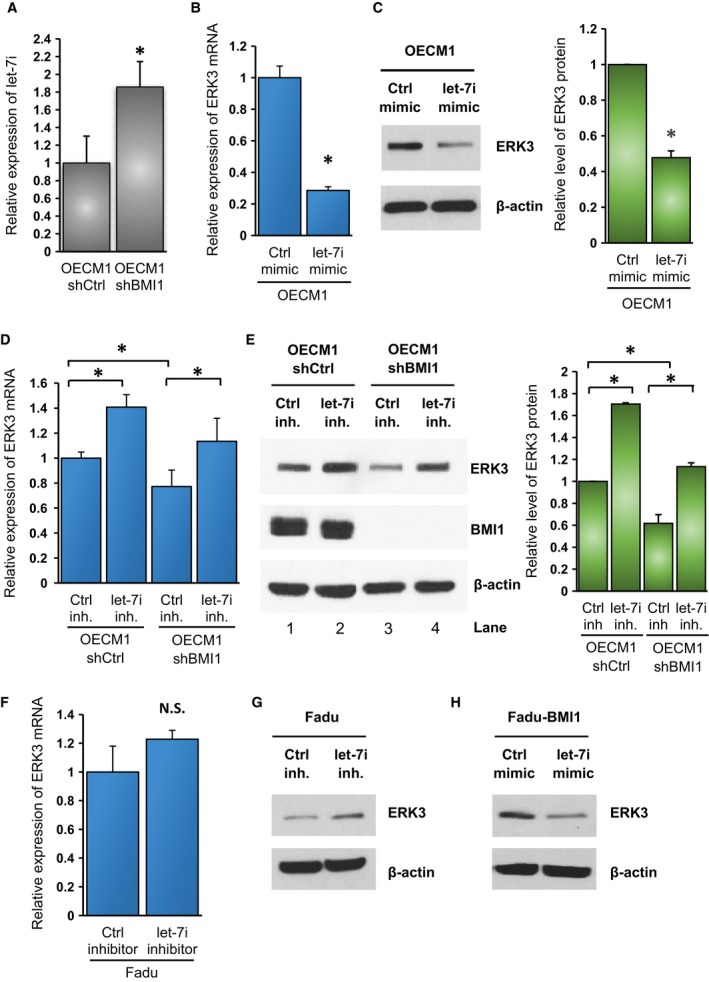
BMI1 upregulates ERK3 expression via miRNA let‐7i. (A) RT‐qPCR analysis of let‐7i in OECM1 cells stably expressing shCtrl or shBMI1. Values in bar graph represent mean ± SD. *P < 0.05 (Student's *t*‐test). (B) RT‐qPCR analysis of ERK3 gene expression in OECM1 cells treated for 2 days with nontargeting control miRNA mimic (Ctrl mimic) or let‐7i mimic (40 nm). The bar graph represents the mean ± SD of three experiments. *P < 0.005 (Student's *t*‐test). (C) Western blot analysis of ERK3 protein level in OECM1 cells treated for 3 days with nontargeting control miRNA mimic (Ctrl mimic) or let‐7i mimic (40 nm). The bar graph in the right panel represents quantification of protein level by densitometry analysis (mean ± SE) of immunoblots of three experiments. The band density in ‘Ctrl mimic’ was set as 1. *P < 0.01 (Student's *t*‐test). (D) RT‐qPCR analysis of ERK3 gene expression in OECM1 stable shCtrl or shBMI1 cells transfected with a nontargeting control inhibitor (ctrl inh.) or let‐7i inhibitor (inh.) at the concentration of 40 nm. Values in bar graphs represent mean ± SD. *P < 0.05 (Student's *t*‐test). (E) Left panel: western blot analysis of ERK3 and BMI1 protein levels in OECM1 stable cells transfected with 40 nm of a nontargeting control inhibitor or let‐7i inhibitor. Right panel: quantification of ERK3 protein level in the immunoblots. The band density in ‘Ctrl inh. of OECM1‐shCtrl’ was set as 1. Data represent mean ± SE. *P < 0.05 (Student's *t*‐test). (F) RT‐qPCR analysis of ERK3 gene expression in Fadu cells transfected with 40 nm of either a nontargeting control inhibitor or let‐7i inhibitor. Values in bar graphs represent mean ± SD of three separate experiments. NS, not significant (Student's *t*‐test). (G) Western blot analysis of ERK3 protein level in Fadu cells transfected with either a nontargeting control inhibitor (ctrl inh.) or let‐7i inhibitor (inh.) at the concentration of 40 nm. (H) Western blot analysis of ERK3 protein level in Fadu‐BMI1 stable cells transfected with a nontargeting control mimic or let‐7i mimic (40 nm).

Next, we wanted to elucidate the molecular mechanism of regulation of ERK3 by BMI1. As BMI1 is a transcriptional repressor but positively regulates ERK3 expression, we postulated an indirect mechanism for this regulation, by which BMI1 suppresses a molecule that downregulates ERK3 expression. miRNAs, negative regulators of gene expression at post‐transcriptional level, would be potential candidates. Interestingly, miRNA let‐7i was shown to be repressed by BMI1 in head and neck cancer cells (Chou *et al*., 2013; Yang *et al*., 2012). In agreement with previous findings, stable knockdown of BMI1 led to increase in let‐7i level in OECM1 cells (Fig. 2A), suggesting that let‐7i may target ERK3 mRNA because knockdown of BMI1 led to decrease in ERK3 expression (Fig. 1). Indeed, treatment of OECM1 cells with let‐7i mimic greatly reduced ERK3 expression at both mRNA and protein levels (Fig. 2B,C). Conversely, suppression of let‐7i by let‐7i inhibitor in OECM1 cells led to increase in ERK3 expression at both mRNA (Bar 2 versus Bar 1, Fig. 2D) and protein levels (Lane 2 versus Lane 1 of ERK3 blot, Fig. 2E). Importantly, while stable knockdown of BMI1 led to decrease in ERK3 protein level (Lane 3 versus Lane 1, Fig. 2E) and increase in let‐7i level (Fig. 2A), suppression of let‐7i by let‐7i inhibitor restored ERK3 protein level under the condition of stable BMI1 knockdown in OECM1 cells (Lane 4 versus Lane 3, Fig. 2E). These results strongly suggest that let‐7i negatively regulates ERK3 expression and that BMI1 upregulates ERK3 expression through suppressing let‐7i expression.

The regulation of ERK3 by BMI1/let‐7i axis was confirmed in Fadu cells. Treatment with let‐7i inhibitor in parental Fadu cells led to increase in ERK3 protein level (Fig. 2G), whereas let‐7i mimic greatly decreased ERK3 protein level in Fadu cells stably overexpressing BMI1 (Fig. 2H). Of note, while treatment with let‐7i inhibitor led to increase in ERK3 expression at both protein and mRNA levels in OECM1 cells (Fig. 2D,E), it increased ERK3 protein level, but had no effect on ERK3 mRNA expression in Fadu cells (Fig. 2F). These results suggest that in OECM1 cells let‐7i facilitates ERK3 mRNA degradation and/or suppresses ERK3 mRNA translation, whereas in Fadu cells, let‐7i may only affect ERK3 mRNA translation. In line with the differential effects of let‐7i on ERK3 expression in different cell lines, BMI1 upregulates ERK3 at both protein and mRNA levels in OECM1 cells (Fig. 1A,B), but only at protein level in Fadu cells (Fig. 1C,D), further suggesting that BMI1 regulates ERK3 expression through targeting let‐7i.

### 3.3 let‐7i downregulates ERK3 expression by directly targeting the 3′UTR of ERK3 mRNA

**Figure 3:**
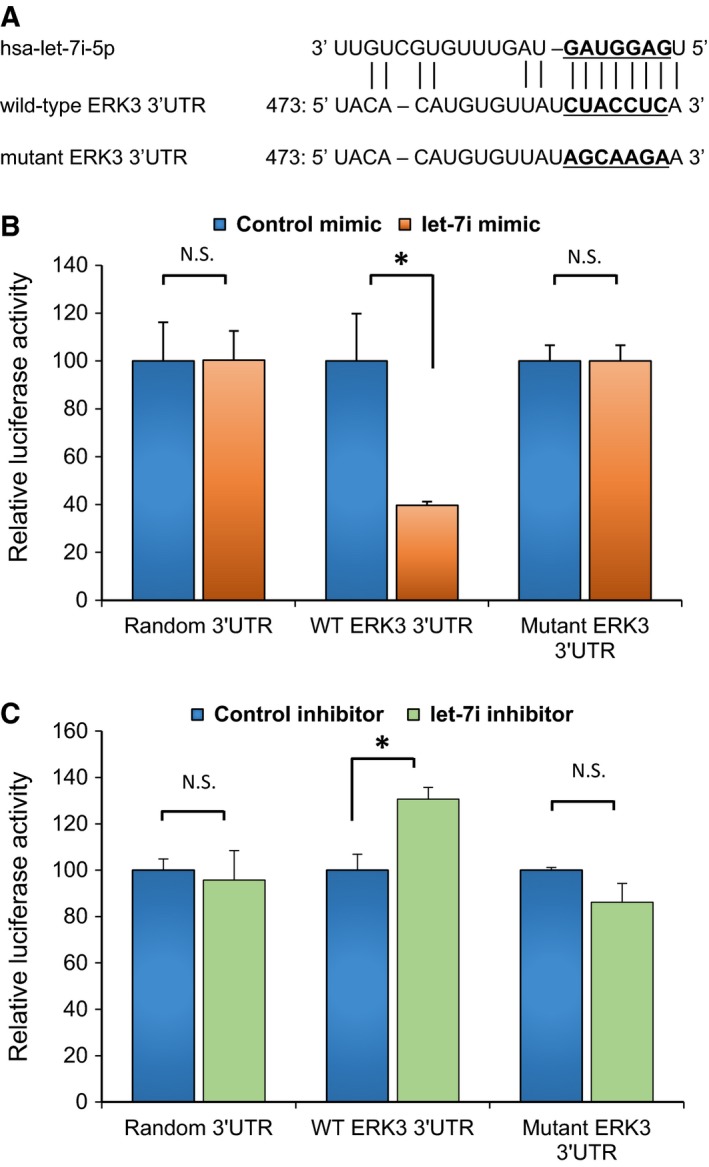
The 3′UTR of ERK3 mRNA is a direct target for let‐7i miRNA. (A) Schematic illustration of the putative let‐7i binding site in ERK3 3′UTR. The let‐7i seed region, the putative let‐7i binding site of ERK3 3′UTR, and the mutated let‐7i binding site were underlined and bold. (B,C) Luciferase reporter assay of HeLa cells cotransfected with a luciferase reporter plasmid (harboring either a random 3′UTR sequence, ERK3 3′UTR, or the mutant ERK3 3′UTR) and a miRNA mimic (a nontargeting control mimic or let‐7i mimic) in (B) or miRNA inhibitor (a nontargeting control inhibitor or let‐7i inhibitor) in (C). Values in bar graphs are the relative luciferase activity (percentage of control mimic or inhibitor) and represent mean ± SE of three independent experiments. *P < 0.05; NS, not significant (Student's *t*‐test).

miRNAs regulate gene expression by binding to the 3ʹUTRs of the targeted mRNAs via partial complementarity (Lee, 2014; Ranganathan and Sivasankar, 2014). Thus, we wanted to determine whether let‐7i regulates ERK3 expression by targeting the 3′UTR of ERK3 mRNA. Indeed, we identified a putative let‐7i binding site in the 3′UTR of ERK3 mRNA (Fig. 3A). Next, we performed a luciferase reporter assay to validate the targeting of the 3′UTR of ERK3 mRNA by let‐7i. As shown in Fig. 3B, while let‐7i mimic did not change the luciferase activity when luciferase gene is followed by a random 3′‐UTR, it greatly reduced the luciferase activity when ERK3 3′‐UTR is present downstream of the luciferase gene, suggesting that ERK3 3′UTR is a target for let‐7i. To confirm this, we generated a luciferase reporter construct in which the ERK3 3′UTR harboring mutations of the let‐7i binding site (shown in Fig. 3A) is placed downstream of the luciferase gene. Importantly, mutation of the let‐7i binding site inhibited the negative regulation of let‐7i mimic on luciferase activity (Fig. 3B). On the contrary, let‐7i inhibitor significantly increased luciferase activity in cells transfected with ERK3 3′UTR luciferase reporter, but had no significant effect on the random 3′UTR luciferase reporter nor the mutant ERK3 3′UTR luciferase reporter (Fig. 3C). These results clearly demonstrate that ERK3 mRNA 3′‐UTR is a direct target of let‐7i.

### 3.4 The BMI1/let‐7i/ERK3 pathway regulates head and neck cancer cell migration

**Figure 4:**
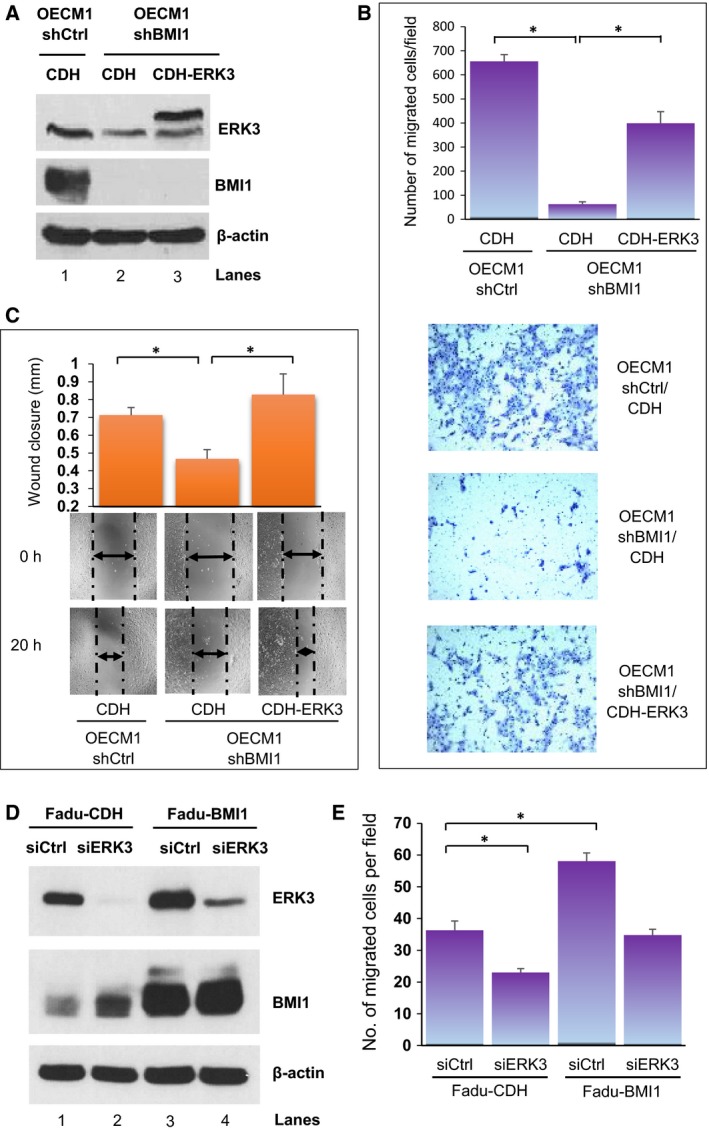
ERK3 is a downstream effector for BMI1‐induced cancer cell migration. (A) Western blot analysis of ERK3 and BMI1 proteins in OECM1‐shCtrl cells and OECM1‐shBMI1 cells that were transiently transduced with either a lentiviral empty vector pCDH‐CMV‐MCS‐EF1‐Puro (CDH) or pCDH‐Myc6‐ERK3 (CDH‐ERK3). (B) Transwell migration assay of OECM1‐shCtrl and OECM1‐shBMI1 cells with transient lentiviral expression of either the empty vector CDH or CDH‐ERK3. Quantitative migration ability under each condition was presented as the number of migrated cells per field (upper panel). Values in bar graph represent mean ± SE. *P < 0.0001 by Student's *t*‐test. Below are representative images of migrated cells stained with crystal violet. (C) Wound healing assay of OECM1‐shCtrl and OECM1‐shBMI1 cells with transient lentiviral expression of either the empty vector CDH or CDH‐ERK3. Wound closure was calculated by subtracting the wound width (mm) at 20‐h time point from the initial wound width at 0 h. Values represent mean ± SD. *P < 0.05 by Student's *t*‐test. (D,E) Fadu cells stably expressing either the empty vector (Fadu‐CDH) or BMI1 (Fadu‐BMI1) were transiently transfected with either a nontargeting control siRNA (siCtrl) or siRNA targeting ERK3 (siERK3), followed by western blot analysis of ERK3 and BMI1 (D) and transwell migration assay (E). Values represent mean ± SE. *P < 0.05 by Student's *t*‐test.

To determine the functional significance of the BMI1/let‐7i/ERK3 pathway in cancer cells, we decided to study the effects of their interplay on cancer cell migration, a cellular function shared by all of them. As expected, stable depletion of BMI1 reduced ERK3 protein level (Lane 2 versus Lane 1, Fig. 4A) and also decreased the migration of OECM1 cells in a transwell migration assay (shBMI1/CDH versus shCtrl/CDH, Fig. 4B). Importantly, restoring ERK3 level by lentiviral expression of ERK3 cDNA (Lane 3, Fig. 4A) rescued the migration ability of OECM1 cells with stable depletion of BMI1 (shBMI1/CDH‐ERK3 versus shBMI1/CDH, Fig. 4B). Similar results were observed in wound healing cell migration assay (Fig. 4C). These results suggest that ERK3 mediates the role of BMI1 in promoting cancer cell migration.

We further corroborate the importance of BMI1/ERK3 axis in cell migration in Fadu cells. In line with previous findings, knockdown of ERK3 (Lane 2 versus Lane 1, Fig. 4D) significantly decreased Fadu cell migration (CDH/siERK3 versus CDH/siCtrl, Fig. 4E). Also, BMI1 overexpression increased ERK3 expression (Lane 3 versus Lane 1, Fig. 4D) and Fadu cell migration (BMI1/siCtrl versus CDH/siCtrl, Fig. 4E). Importantly, knockdown of ERK3 suppressed the increase in cell migration induced by BMI1 overexpression (BMI1/siERK3 versus CDH/siCtrl, Fig. 4E), further suggesting that ERK3 is a critical downstream mediator of BMI1 in promoting cell migration.

**Figure 5:**
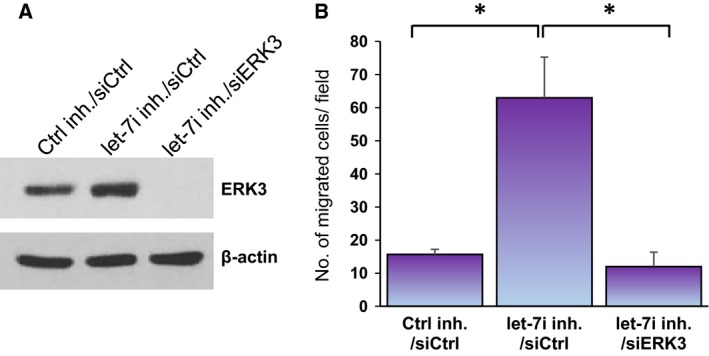
Suppression of let‐7i by let‐7i inhibitor greatly increases migration of OECM1 cells with stable BMI1 knockdown and ERK3 depletion inhibits this effect. OECM1‐shBMI1 cells were cotransfected with a control inhibitor (Ctrl inh.) or let‐7i inhibitor (let‐7i inh.) and with either a nontargeting control siRNA (siCtrl) or siRNA against ERK3 (siERK3), followed by western blot analysis of ERK3 (A) or a transwell migration assay (B). Values represent mean ± SD. *P < 0.001 (Student's *t*‐test).

As let‐7i level was shown to be increased concomitant with decrease in ERK3 expression when BMI1 was stably knocked down in OECM1 cells (Fig. 1A and Fig. 2A), we anticipated that treatment with let‐7i inhibitor would restore ERK3 protein level and cell migration ability of OECM1‐shBMI1 cells. Indeed, both ERK3 protein levels (Fig. 5A) and cell migration ability (Fig. 5B) were greatly increased when OECM1‐shBMI1 cells were treated with let‐7i inhibitor (compare let‐7i inh./siCtrl with ctrl inh./siCtrl). Notably, knockdown of ERK3 (Fig. 5A) inhibited the increase in cell migration induced by the treatment with let‐7i inhibitor (compare let‐7i inh./siERK3 with ctrl inh./siCtrl, Fig. 5B), suggesting that let‐7i suppresses OECM1 cell migration by downregulating ERK3. Taken together, the results in Figs 4 and 5 clearly demonstrate the importance of the BMI1/let‐7i/ERK3 pathway in regulating head and neck cancer cell migration: Both BMI1 and ERK3 promote migration and let‐7i, as an intermediator for BMI1 and ERK3, inhibits cell migration.

### 3.5 ERK3 and BMI1 protein levels positively correlate in head and neck cancer tissues

**Figure 6:**
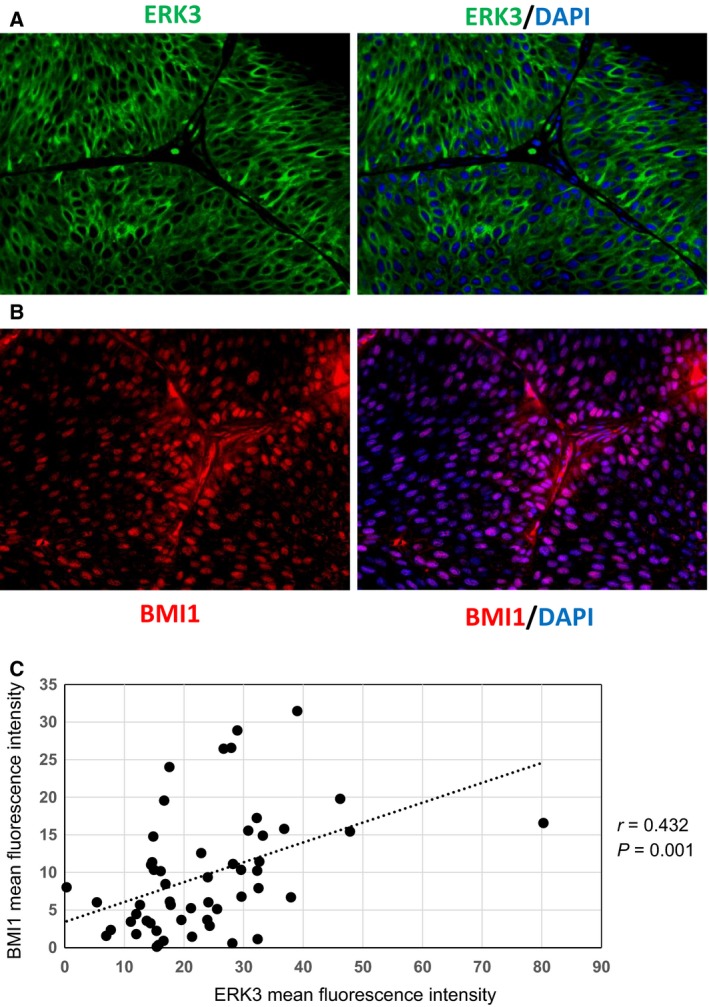
BMI1 and ERK3 protein levels positively correlate in head and neck cancer tissues. (A,B) Representative images for immunofluorescent staining of ERK3 (A) and BMI1 (B) in FFPE head and neck tumor specimens. Cells’ nuclei were marked by DAPI staining. The merged images are shown on the right. Pictures were taken at 200 × magnification. (C) Scatter plot for the correlation between ERK3 expression and BMI1 expression in head and neck cancer tissues with stages I to IV and metastatic lesions. ‘*r*’ stands for Pearson's correlation coefficient and *P* = 0.001.

To investigate the clinic relevance of our finding on the regulation of ERK3 expression by BMI1, we examined the levels of both ERK3 and BMI1 proteins by immunofluorescent staining in a FFPE head and neck cancer tissue microarray which contains tumor specimens with stages I to IV from totally 68 patients. ERK3 staining was primarily localized in the cytoplasm (Fig. 6A), whereas BMI1 was mainly observed in the nucleus (Fig. 6B). In agreement with our finding in cultured HNSCC cells that ERK3 is upregulated by BMI1, there is a positive correlation between ERK3 level and BMI1 level in the head and neck cancer tissues (correlation coefficient *r* = 0.432, *P* = 0.001) (Fig. 6C).

**Figure 7:**
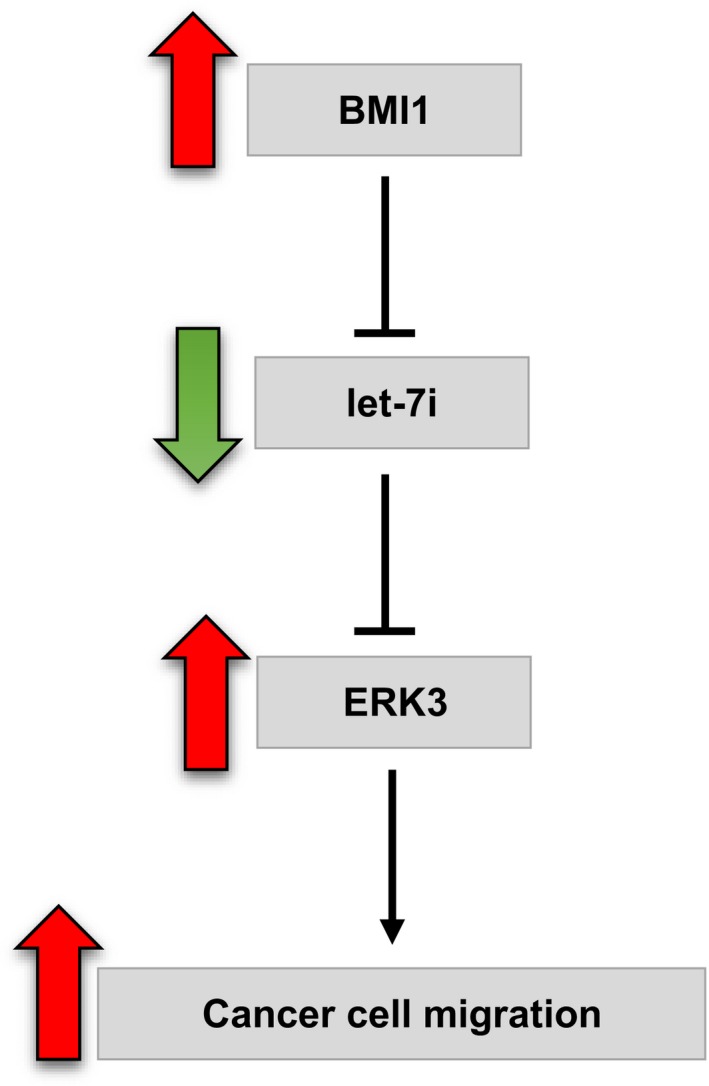
A schematic model for the BMI1/let‐7i/ERK3 pathway in controlling cancer cell migration. This model presents the regulation of ERK3 expression by BMI1 and let‐7i: ERK3 mRNA is a direct target of let‐7i miRNA; elevation of BMI1 (indicated by the upward arrow) suppresses let‐7i (Chou *et al*., 2013; Yang *et al*., 2012) and upregulates ERK3, leading to increase in head and neck cancer cell migration.

Based on the results of this study, we propose a novel molecular mechanism for the upregulation of ERK3 in cancer: BMI1 suppresses the transcription of the miRNA let‐7i, a negative regulator of ERK3 expression, which leads to elevation in ERK3 protein level and increase in cancer cell migration (Fig. 7).

## 4 Discussion

ERK3, as a member of the atypical MAPK subfamily, was cloned 25 years ago together with ERK2 by homology screening of a rat brain cDNA library using an ERK1‐derived probe (Boulton *et al*., 1991). While the conventional MAPKs, such as ERK1/2, have been extensively studied, much less is known about the molecular regulation of ERK3 activity and expression. ERK3 has gained attention in recent years due to the discovery of its important physiological and pathological functions. Mice with homologous deletion of ERK3 gene show neonatal lethal phenotype due to severe defect in lung differentiation and maturation (Klinger *et al*., 2009). ERK3 is also an important regulator for thymocyte activation and survival (Marquis *et al*., 2014a,b; Sirois *et al*., 2015), neuronal morphogenesis (Brand *et al*., 2012), and endothelial cell migration (Wang *et al*., 2014). As described in the 1, the importance of ERK3 in cancers has also been revealed. Although ERK3 is known to be upregulated in multiple cancers and promotes cancer cell migration and invasion, the regulation of ERK3 expression in cancer cells is largely unknown except for one study showing the positive regulation of ERK3 by BRAF in melanoma cells (Hoeflich *et al*., 2006).

Here, we reveal a new molecular mechanism for regulating ERK3 expression in head and neck cancer cells. Our finding about the tight regulation of ERK3 expression by the BMI1/let‐7i axis is important given that BMI1 is a well‐known oncoprotein and that let‐7i is a tumor suppressor in multiple cancers including head and neck cancers. BMI1 protein is a key regulatory component of PRC1 transcription suppressor complex (Cao *et al*., 2011). Overexpression of BMI1 has been detected in several human cancers including head and neck cancer, colorectal carcinoma, non‐small‐cell lung cancer, and prostate cancer (Cao *et al*., 2011). Numerous studies have shown that BMI1 promotes cancer cell growth and migration/invasion, but inhibits apoptosis (Cao *et al*., 2011; Chou *et al*., 2013; Siddique and Saleem, 2012; Song *et al*., 2009; Yang *et al*., 2012). It acts as an oncoprotein through suppressing tumor suppressor genes including p16Ink4a, p14Arf, PTEN, and miRNAs (Cao *et al*., 2011; Jacobs *et al*., 1999; Song *et al*., 2009). In addition, BMI1, through indirect mechanisms, can upregulate genes that promote cancer progression, such as the kinase Aurora A (Chou *et al*., 2013), and several genes involved in epithelial‐to‐mesenchymal transition and transforming growth factor‐β and epidermal growth factor/platelet‐derived growth factor pathways (Ferretti *et al*., 2016). Our study identified ERK3 as a new target of BMI1 that is critical for cancer cell motility. Restoring ERK3 level by exogenous expression in cells with BMI1 depletion rescued cancer cell migration, while depletion of ERK3 in BMI1‐overexpressing cells greatly diminished the effect of BMI1 promoting cancer cell migration, demonstrating that ERK3 functions downstream of BMI1 in promoting head and neck cancer cell motility. More importantly, we show that the levels of these two proteins positively correlate in HNSCC tumor specimens, highlighting the clinical significance of our study.

Let‐7i is a member of the let‐7 family of tumor suppressor miRNAs that inhibits cancer cell proliferation, survival, migration, and invasion by downregulating a variety of oncogenes such as myc, Ras, HMGA2, and Lin28B (Boyerinas *et al*., 2010). Downregulation of let‐7 miRNAs leads to upregulation of oncogenes and promotes tumor initiation and progression. As such, they can serve as diagnostic and/or prognostic tumor markers (Monroig‐Bosque Pdel *et al*., 2015). More importantly, let‐7 miRNAs have been utilized for suppressing tumor growth and progression in animal tumor studies, underscoring their therapeutic use for treating cancers. For example, delivery of let‐7b or let‐7g either by adenoviral infection or by nanoparticles successfully inhibited lung tumor progression in both xenograft and transgenic tumor mouse models (Kasinski *et al*., 2015; Kumar *et al*., 2008; Trang *et al*., 2010). In comparison with let‐7b and let‐7g, the therapeutic potential of let‐7i in cancers is largely unexplored. Our study identified ERK3 as a new target of let‐7i and reveals that the let‐7i/ERK3 axis plays an important role in controlling head and neck cancer cell motility. Interestingly, the let‐7i/ERK3 axis is tightly controlled by BMI1, an oncoprotein well known to be important for tumor growth and recurrence, as well as metastasis. Hence, our study not only provides better understanding of let‐7i's tumor suppressing roles and the underlying mechanisms but also substantiates the importance of targeting BMI1 and let‐7i for treating cancers. In addition, our findings suggest that ERK3 kinase is a potential new therapeutic target of cancers, particularly those with BMI1 overexpression.

## Author contributions

The experiments were designed by WL and LE. LE carried out the experiments and data analysis for Figs 1, 2, 3, 4, 5, 6, except for Figs 1C and 4A,C,E which were conducted by MC. KM examined and evaluated both H&E and immunofluorescent slides of head and neck tumor specimens. MY developed OECM1‐shCtrl and OECM1‐shBMI1 cell lines and participated in interpreting the data. The manuscript was written by LE and WL with inputs and comments from all coauthors. All authors have read and approved the final version of the manuscript.
